# Meteorological Variables Associated with Stroke

**DOI:** 10.1155/2014/597106

**Published:** 2014-11-30

**Authors:** Romy Nocera, Philip Petrucelli, Johnathan Park, Eric Stander

**Affiliations:** Department of Emergency Medicine, Drexel University College of Medicine, MS 1011, NCB, 245 N. 15th Street, Philadelphia, PA 19102, USA

## Abstract

To elucidate relationships between meteorological variables and incidence of stroke, we studied patients diagnosed with stroke after presenting to the emergency department (May 1, 2010–August 8, 2011). Patient demographics and medical data were reviewed retrospectively with regional meteorological data. Across 467 days, 134 stroke events were recorded on 114 days. On stroke days, maximum temperature (max *T*) and atmospheric pressure (AP) combined were a significant predictor of stroke (max *T* odds ratio (OR) = 1.014, 95% confidence interval (CI) = 1.003–1.026, and *P* = 0.04; AP: OR = 1.033, 95% CI = 0.997–1.071, and *P* = 0.02). When the patient could identify the hour of the stroke, average temperature (avg *T*) was significantly higher than nonstroke hours (18.2°C versus 16.16°C, *P* = 0.04). Daily fluctuations in AP and avg *T* also had significant effects on stroke incidence (AP: OR = 0.629, 95% CI = 0.512–0.773, and *P* = 0.0001; avg *T* OR = 1.1399, 95% CI = 1.218–606, and *P* = 0.0001). Patient age, stroke history, body mass index, ethnicity, and sex were further contributors to stroke risk. Temperature, atmospheric pressure, and certain physiological conditions likely play roles in weather-related stroke susceptibility. The mechanisms driving these associations are not fully understood.

## 1. Introduction

Stroke continues to be a leading cause of morbidity and mortality, with clearly identified physiological and lifestyle risk factors. Weather conditions may also be connected to stroke, and multiple meteorological variables have been examined as possible influences on stroke occurrence. Across studies, findings are inconsistent, complex, and often contradictory and are further confounded by stroke subtype, physiological risk factors, and interactions between variables.

Atmospheric pressure (AP) and temperature are among the most closely studied variables; increases, decreases, and fluctuations in both have been significantly linked to numerous stroke subtypes. Incidence of subarachnoid hemorrhage (SAH) has been correlated with increased absolute AP [1, 2], with change in AP from the previous day [1, 3, 4], and notably with a change of >5 hPa [3]. AP change of >10 hPa within 24 hours was found to be associated with SAH clusters (>2/day) and with cluster days and consecutive (series) days of events impacted by maximal AP difference 24 hours prior to the event [4]. Lejeune et al. (1994) found a 2% increase in SAH was attributed to a 100 hPa reduction in AP [5]. A 6.6% increase in hemorrhagic stroke was also observed per 10 hPa pressure decrease within 48 hours prior to the stroke event [6], and a fall of >73 hPa in the preceding 72 hours was associated with clusters of male patients hospitalized for SAH [7]. Risk of nonlacunar ischemic stroke increased almost 4-fold when AP fell more than 3 hPa from the previous day [8], and increased emergency admissions due to ICH were observed for every 1 hPa drop in AP from the day prior to the event [9]. Conversely, another study found that ICH incidence was associated with a rise in AP [8].

Studies of the role of ambient temperature on stroke risk are likewise inconsistent. Decreased temperature has been related to increased incidence of ischemic stroke (IS) 24 to 48 hours after exposure to cold weather [10]. An average 5°C fall in mean temperature was associated with a 7% elevation in hospital admissions of young women for IS [11]; similarly, low average ambient temperature and number of cold days per year correlated with higher risk of total stroke incidence in female patients, independent of other risk factors [12]. A 1°C drop in minimum temperature from the previous day was associated with a 3.9% increase and 5.0% increase in IS and cardioembolic IS, respectively; relative risk for a fatal versus a nonfatal stroke increased by 15.5% for a 1°C drop in maximum temperature in the same period [13]. Decreases in maximum, minimum, and average temperatures have also demonstrated significant relationships to increased clustering of ischemic events [14].

Low temperature the day before and the day of stroke onset has also been found to be a significant risk factor for SAH [2, 5] and ICH [15]. Ohwaki et al. (2004) noted that days on which hypertensive ICH occurred had significantly lower minimum temperatures and decreased minimum temperatures compared with the previous day, with ICH being most frequent when minimum temperature was <5°C [15]. Furthermore, incidence of primary ICH increased by 11.8% for each degree drop in the diurnal temperature range from the day before the event [13]. Similarly, a 1°C lower average temperature over the same day and the previous 4 days was associated with a 2.7% higher admission rate for hemorrhagic stroke, especially among women and older subjects [16].

A significant effect of higher temperature on stroke has also been reported. For example, every 1°C increase in mean temperature during the preceding 24 hours was associated with a 2.1% rise in hospital admissions for IS [6]. Hospitalizations for both hemorrhagic and ischemic strokes were low when ambient temperature at 6, 12, and 24 hours prior to initial symptoms was <20°C and peaked at temperatures between 23°C and 24°C [17]. Mortality due to IS correlated with a 1°C increase in mean temperature, with an estimated percentage change in mortality above a threshold ranging from 2.3% and 5.4%, although risk decreased after adjustment for confounders [18].

As with AP, stroke occurrence may be influenced by temperature fluctuations rather than absolute levels. Changes of plus or minus ≥5°C over 24 hours were related to and highly predictive of an increased risk of acute ischemic and other types of stroke, especially among persons already at moderate risk [19] and in patients older than 65 years [20]. For cases of primary ICH, sudden increased risk of hospitalization occurred when changes in temperature exceeded ±5°C [20]. Coelho et al. (2010) found that, for both hemorrhagic and ischemic stroke, during the 24 hours before the event almost all patients experienced initial stroke symptoms within a comfortable temperature range but after a change of 3°C [17]. Days showing clusters of SAH differed significantly in maximum difference in daily temperature compared with noncluster days [4], and in a 7-day lag analysis, a decreasing change in maximum temperature was associated with an increase in stroke [21].

Additional meteorological variables appear to influence stroke incidence, either individually or in aggregation. Decreases in several measures of humidity on the day of and/or day before stroke have been significantly linked to individual and clustered IS and transient ischemic attack [15], individual SAH events [5], and both clusters and series days of SAH [4]. In a study of multiple vascular diseases, days on which cerebral infarction occurred correlated with more humidity factors, fewer sunshine hours, fewer solar radiation factors, and greater amounts of precipitation factors [22]. Among women, high annual rainfall, low average ambient temperature, and number of cold days per year were associated with increased risk of stroke incidence independent of risk factors [12]. Primary ICH increased on days with a 1 mm/m^2^ drop in precipitation and an 11.8% drop in diurnal temperature [13], and in men, changes in precipitation, falling AP, dropping dew point temperature, or a combination of these occurred 72 hours prior to clusters of SAH [7]. Subarachnoid hemorrhage has beenfound to increase with decreased sunshine duration, temperature, and humidity on the day of onset and decreased temperature, AP, and humidity on the day before onset [5].

Despite such evidence of the impact of climatic factors on stroke, several studies report no relationship between AP and the incidence of particular stroke subtypes [15, 23] and no direct influence of temperature on stroke of any subtype [8], for any age or either sex [24], or during any season [24–26]. Another research has shown no direct relationship between humidity and stroke, including SAH [2, 25], IS [8, 11, 13, 24], hemorrhagic stroke [16], and ICH [8, 13, 15], or between duration of sunlight and stroke or cardiovascular disease [15, 16, 24, 27].

The relationship between meteorological factors and stroke is highly complex and influenced by numerous other factors. Variability in study design has contributed to inconclusive results, and many studies have neglected to include relevant factors and confounders. The purpose of this study was to further describe and clarify the relationship between weather and stroke, by including numerous patient characteristics and meteorological variables in order to identify singular and interactive influences of weather variables on stroke.

## 2. Method

### 2.1. Design

This retrospective chart review was conducted at a large urban university hospital. Prior to data collection, the study was approved by the university's medical institutional review board. As this was a retrospective study, informed consent was not required. Data were collected over 467 days, from May 1, 2010, to August 8, 2011. Stroke subjects were identified via weekly reports from the hospital stroke program coordinator. Patients were considered eligible for the study only if they had presented at the hospital's emergency department. This department provides 24 hours, 7 days a week care, with complete diagnostic and testing methods and access to the neurology department stroke team. Stroke patients who were admitted directly from their doctor's office, from a rehabilitation center, or from other sources were not included in the study. For eligible patients, stroke was defined as per ICD-9 codes. For each stroke patient, the Discharge Summary and Coding Summary were carefully reviewed to ensure that the patient did indeed have a stroke (of any type) as diagnosed by a board-certified neurologist. Patients were only included if they had a complete diagnostic workup and received a diagnosis of stroke, with accompanying ICD-9 code. Patient charts were viewed in a secure electronic document management system by the principal investigator and two masters-level graduate research assistants. All reviewers trained together to ensure consistency and reliability of data collection. Data were recorded for multiple demographic, medical, and physiological factors (Table 1). For physiological factors that were measured more than once, the first reading taken after the patient presented to the emergency department was documented. Body mass index (BMI) was determined using the NIH BMI calculator. Meteorological data were collected online from the National Climatic Data Center and from the Pennsylvania State Climatologist (Table 2).

### 2.2. Statistical Analyses

Data were analyzed using Excel (Microsoft, Redmond, WA) and XLStat (Addinsoft, New York, NY) programs. Logistic regression, *t*-tests, and correlation analyses were used to determine the influence of meteorological variables on incidence of stroke and the further impact of demographic and physiological characteristics of stroke patients in the context of weather conditions. For all analyses, *P* value was set as ≤0.05.

## 3. Results

**Figure 1:**
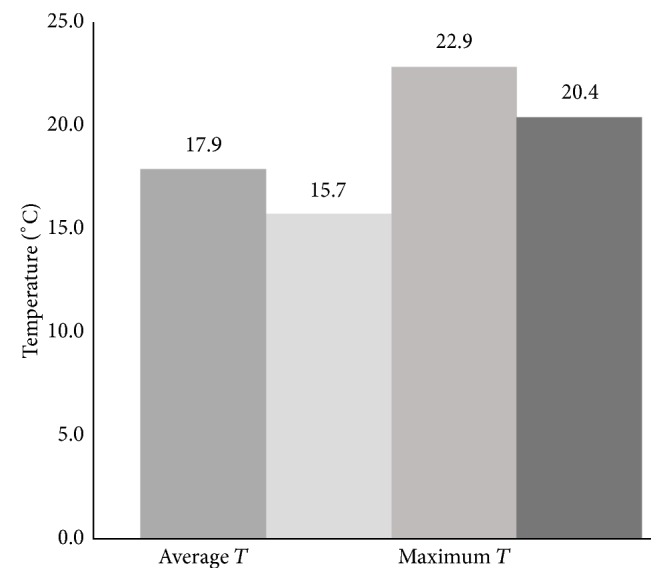
Differences in temperature (°C) on stroke days versus nonstroke days.

**Figure 2:**
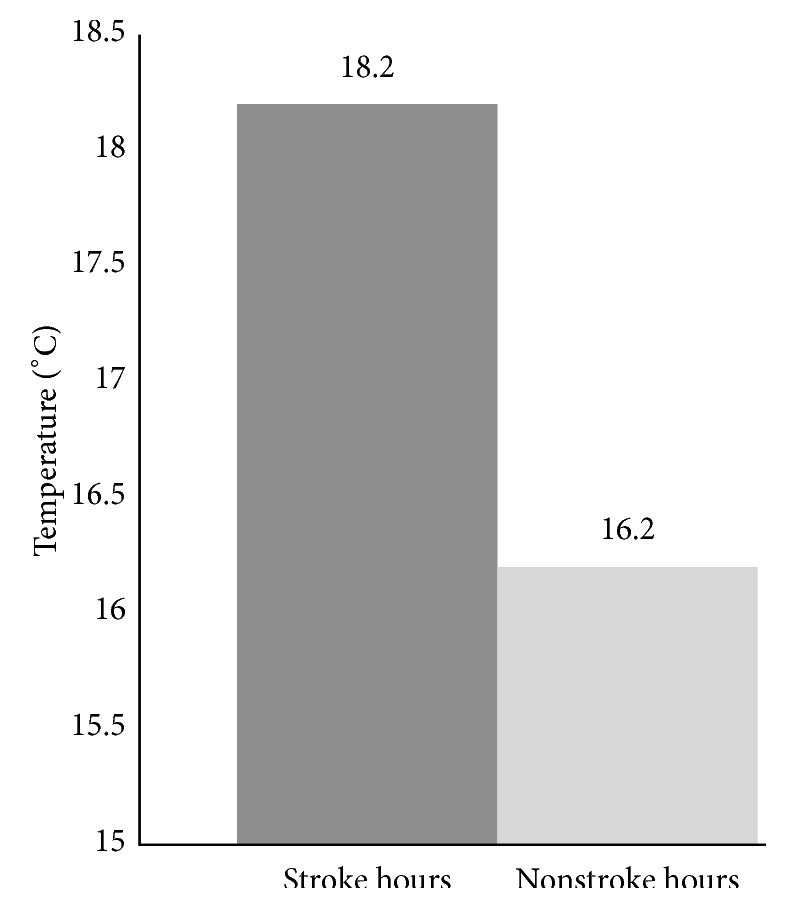
Difference in avg *T* during stroke hours versus nonstroke hours.

Strokes occurred on 114 days for a total of 134 stroke events. Based on International Classification of Disease (9th rev.) coding, the majority (93%) were ischemic. Demographic information on the stroke patients is presented in Table 3. The 7 meteorological variables were compared between stroke days and nonstroke days. Data were entered into forward logistic regression, with the outcome variable as stroke versus nonstroke days. While some variables were removed by the regression model, others demonstrated significant impact on stroke incidence. In the regression analysis, maximum temperature (max *T*) and AP combined were a significant predictor of stroke (max *T*: odds ratio (OR) = 1.014, 95% confidence interval (CI) = 1.003–1.026, and *P* = 0.04; AP: OR = 1.033, 95% CI = 0.997–1.071, and *P* = 0.02). Max *T* was significantly higher on stroke days than on nonstroke days (73.3°F versus 68.8°F (22.9°C versus 20.4°C), *P* = 0.04; Figure 1). When all meteorological variables were entered into logistic regression, average temperature (avg *T*) was initially removed from the model based on multicollinearity; *t*-test showed significantly higher avg *T* on stroke than on nonstroke days (64.3°F versus 60.2°F (17.9°C versus 15.7°C), *P* = 0.04; Figure 1). A nonsignificant trend was also found for a higher minimum temperature on stroke days than on nonstroke days. The majority of patients (*n* = 112) were able to identify the specific time, within an hour, when their stroke occurred. Avg *T* at times of stroke events was significantly higher than avg *T* during hours in which a stroke did not occur (64.77°F versus 61.09°F (18.2°C versus 16.2°C), *P* = 0.04; Figure 2). Average AP did not differ between stroke and nonstroke hours.

Seventeen days had multiple stroke occurrences. The maximum number of strokes on a single day was 3. Two days had 3 stroke events; one of these was the hottest day in our sample (max *T* = 105.1°F (40.6°C)). Meteorological factors on days with multiple strokes were compared with meteorological factors on all other days combined (0 or 1 stroke), days with 1 stroke, and days with no strokes. In these comparisons, days with multiple strokes showed higher max *T*, min *T*, avg *T*, and hours of sunlight than days with 1 or 0 strokes, although no differences were statistically significant.

There were more hours of sunlight on stroke days than on nonstroke days (12.8 versus 12.4, *P* = 0.05). This variable correlated strongly with avg *T* (*r* = 0.84, *P* < 0.0001); therefore we examined hours of sunlight on days when avg *T* was relatively cool, defined as less than the median of all days (66°F (19°C)). We found only a nonsignificant trend for more hours of sunlight on stroke days than on nonstroke days; thus it is unlikely that duration of sunlight itself was significantly related to stroke incidence. We found no significant relationships between stroke and humidity or precipitation.

We also examined the influence of fluctuations in avg *T*, max *T*, or AP, with changes assessed from 48 and 24 hours prior to day of stroke or no stroke and change (range) on the event day itself. We found significant differences in daily change for AP on stroke versus nonstroke days (OR = 0.629, 95% CI = 0.512–0.773, and *P* < 0.0001) and avg *T* (OR = 1.1399, 95% CI = 1.218–1.606, and *P* < 0.0001). The mean daily change in avg *T* was higher on stroke days than on nonstroke days 17.75°F versus 16.98°F (−7.92°C versus −8.34°C). As indicated by OR of less than 1.00, AP daily change was lower on stroke days than on nonstroke days (6.27 versus 6.75 hPa), with increased likelihood of stroke corresponding to decreasing AP fluctuations. We found no influence of change in AP, avg *T*, or max *T* from 48 or 24 hours to day of stroke or no stroke.

We also analyzed the data to determine relationships between weather factors and patients' demographic, medical, and physiological variables. Two-tailed *t*-tests identified an age effect, in that patients aged 64 years or younger experienced their stroke event at significantly higher avg *T* (20.29°C versus 16.81°C, *P* = 0.04) and min *T* (15.45°C versus 11.70°C, *P* = 0.02) than older patients. For those demographic and physiological factors dichotomized as present/absent, point-biserial correlations assessed whether patients with various characteristics or medical conditions experienced their stroke during distinct meteorological conditions compared with patients without those characteristics or conditions (Table 4). Subjects with a history of stroke experienced their event on days with significantly higher AP than did those with no stroke history (1017.28 versus 1015.05 hPa, *P* = 0.04). Significance tests of frequencies showed that among patients with stroke history, more experienced their stroke above the median average AP than below it (*n* = 34 versus *n* = 14, *P* < 0.0001), as did patients with active hypertension (*n* = 61 versus *n* = 41, *P* = 0.003). Significantly more patients with a normal BMI (18.5–24.9) experienced stroke above both the median AP and median avg *T* than below (*n* = 18 versus *n* = 8, *P* = 0.003). These relationships were not found among stroke patients with BMI classified as overweight (25–29.9) or obese (≥30). There were more African American patients above the avg *T* median than below (*n* = 51 versus *n* = 36, *P* = 0.02). African American and white patients showed nonsignificant trends toward more persons having stroke above the AP median. Our sample did not have enough patients of other ethnicities to include in analyses. Female patients had higher stroke frequency above the median avg *T* than below (*n* = 44 versus *n* = 31, *P* = 0.03); male patients showed a nonsignificant trend in the same direction. Male patients also had a higher stroke frequency above the median AP than below (*n* = 38 versus *n* = 20, *P* = 0.04).

We examined influence of weather factors with regard to severity of stroke, as measured by NIH Stroke Scale scores, length of hospital stay, and disposition upon discharge (sent home, transferred to a rehabilitation facility, or died). We found no relationship between meteorological conditions and scores on the NIH Stroke Scale (NIHSS). Length of hospital stay showed a small negative association with relative humidity (*R* = −0.173, *P* = 0.04). We found no differences in weather factors between patients discharged to home (*n* = 76) compared with those discharged for rehabilitation (*n* = 52). There were more hours of sunlight on the day of stroke for patients who died (*n* = 6) than for those discharged for rehabilitation (14.23 versus 12.46, *P* = 0.02).

## 4. Discussion

Our primary objective was to further explore and clarify the impact of meteorological variables on stroke incidence. Overall, increased temperature was the strongest individual predictor of stroke. This was true of both average and maximum temperature values and also specific times of stroke events. This finding supports previous findings that higher temperatures are associated with stroke [6, 17, 18, 28]. In addition, days with multiple strokes showed higher maximum, minimum, and average temperatures and hours of sunlight than days with 1 or 0 strokes, although no differences were statistically significant. However, the small number of multistroke days likely does not provide enough power for this analysis. Furthermore, the role of sunlight is difficult to determine as this variable had a strong correlation with temperature. Other research found a significant relationship between lower levels of sunlight radiation and higher stroke incidence; however, in this study insolation and temperature levels were also strongly correlated [29].

In contrast to other studies [2, 5, 10–16], we did not find a relationship between cold temperatures and stroke, nor was there a direct effect of increased [1, 2, 8] or decreased [5–7] AP on stroke. The influence of AP appeared to be in conjunction with max *T*, as AP did not significantly predict stroke versus nonstroke days when entered as the sole variable in a logistic regression analysis, nor did average AP differ on stroke versus nonstroke days as determined by *t*-test. Some combinations of AP and patient characteristics and physiological conditions were related to stroke. Invariably these involved higher AP (above median level). This finding lends support to studies describing a definitive relationship between increased AP and stroke.

Numerous studies have reported that fluctuations in AP [1, 3–5] and temperature [4, 17, 19–21] affect stroke incidence. Our findings do not support earlier findings regarding change in these factors 1 or 2 days before the stroke event. As noted, we found a significant change in AP on the stroke day itself. Specifically, daily change in AP in a decreasing direction, combined with rising change in temperature, was associated with increased risk. This finding is difficult to interpret, given that patients experienced their stroke events throughout the 24-hour daily cycle. It is possible that ongoing shifts in these parameters as they continually fluctuated throughout the day may have contributed to the stroke events.

More people with a normal BMI experienced their stroke above median average temperature than below; persons without the increased risk of stroke due to obesity may be particularly susceptible to the influence of temperature. The reason for this is unknown. Among these subjects, none were engaging in any behavior or activity more strenuous than walking at the time of their stroke; therefore it does not seem likely that overexertion in warmer temperatures played a role. Similarly, our age findings may reflect the fact that younger people were more likely to be employed and under greater stress, a known risk factor for stroke. Younger people may also be more active outdoors and thus more susceptible to weather factors.

We found no weather factors related to severity of stroke as quantified by NIHSS score. Importantly, the NIHSS was not administered to all patients immediately upon their arrival in the emergency department; as stroke symptoms can worsen or resolve over time, NIHSS scores recorded at times after the patient's arrival may not reflect the initial severity. Relationships between weather factors and length of hospital stay and disposition at discharge were relatively weak (notably, a low inverse correlation between humidity and length of stay and more hours of sunlight on the stroke day of deceased patients). This finding is not robust given the small number of subjects.

The reasons for associations between weather factors and stroke risk remain to be clearly elucidated. Evidence suggests that several physiological mechanisms may be involved. Temperature may affect sympathetic and hematologic variables such as serum fibrinogen and blood viscosity and may increase nocturnal blood pressure levels [30]. High temperature has been associated with worsening of endothelial function [31] and may elevate risk of dehydration and thus increase blood viscosity and risk of vascular events [32]. Hot weather causes dehydration because of excessive evaporation and sweating, which can lead to electrolyte imbalance, thermoregulatory failure, or thromboembolism [33–35]. Cerebral ischemia is worsened by elevated body temperature in both laboratory animals and human stroke patients [36, 37], and exposure to heat has been associated with increased body temperature in both healthy subjects and persons at risk for thrombosis [38]. The effect of high environmental temperatures on stroke incidence may therefore be via increased body temperature. We did not have information on our subjects' body temperatures at the precise time of stroke; future data collection may include the patient's first recorded temperature in the emergency department.

That our results did not uphold those of some previous research is not surprising. The current literature shows great variability in study design, which meteorological variables were included, how they were defined and measured, consideration of additional physiological factors and other patient characteristics associated with stroke risk, analysis by subtype of stroke, examination for interactions between variables, and sample size. Findings are thus inconsistent and contradictory. Lim et al. [33] also warned against assuming a linear relationship between any meteorological variable and stroke risk, citing studies that failed to find a significant relationship because risk was estimated based on the assumption of linearity. Furthermore, associations between weather and different types of stroke have been described; in our study only 9 (7%) of strokes were hemorrhagic, so we were unable to compare this subclass of stroke versus ischemic.

With such complex relationships between stroke factors and physiological characteristics, it is difficult to develop a profile of persons who will be more at risk for stroke during specific meteorological conditions. In a study of associations between extreme temperatures and mortality, Medina-Ramón et al. [39] found that being 65 years of age or older, diabetic, and African American was related to susceptibility to extreme heat. According to our data, being under age 64 years, having a normal BMI, being African American, and being female were all significantly related to experiencing stroke at higher temperatures. Having a history of stroke, having hypertension, being male, and having a normal BMI were related to having an event at higher AP levels. However, we are unsure of how precisely these factors combine to predispose an individual to stroke.

Understanding how weather factors influence stroke is crucial to increasing awareness of stroke symptoms under particular meteorological conditions and thus potentially preventing stroke. Persons predisposed to stroke or persons with specific physical and medical conditions could be mindful of weather-associated risks. Stroke prevention education could include encouraging people to be alert to signs and symptoms of stroke under certain weather conditions. Increased attention to stroke symptoms may lead to more rapid medical attention, thus limiting damage and preventing a more serious occurrence in the future. Furthermore, emergency departments could be properly equipped and staffed for days when patient load might be increased owing to weather conditions.

This study has several limitations. Only one hospital site was included; stroke patients were undoubtedly being seen in the other emergency departments in the greater metropolitan area. There were likely more stroke patients at the hospital during our period of collection; however, we only included patients who presented at the emergency department. We also excluded those patients who could not provide information regarding the date of stroke onset. Critical data points, including time and date of first stroke symptoms, were sometimes based solely on patient self-report and were thus potentially unreliable. Similarly, several physiological data points may not be entirely accurate, as they were taken at the time of emergency department arrival rather than at the time of the stroke event. For example, hypertension is a known risk factor for stroke and patients with untreated hypertension showed a 3.6-fold increase in ICH risk during the colder months of November to April, with no other variables associated with ICH incidence [40]. We recorded the first blood pressure reading taken once the patient was in the emergency department, which may have differed from blood pressure at time of the event. Therefore our finding of no relationship between blood pressure and weather factors may be inaccurate. Additionally, all meteorological measurements referred to outdoor temperature. It is possible that the indoor ambient temperature differed from that outside, especially during very cold or very hot days. We are unsure whether this difference had an influence, as strokes occurred both indoors and outdoors. We were also unable to compare hemorrhagic and ischemic stroke groups, as there were too few in the former for statistical analysis. Finally, as we found multiple nonsignificant trends, more data will be needed to determine whether these relationships between weather variables and stroke are significant.

## 5. Conclusion

This study further underscores the complex nature of the effect of meteorological factors on stroke incidence. Our data support an association between stroke and increased temperature, daily changes in average temperature and AP, and combined effects of maximum temperature and AP. We also identified age effects and combined effects of some physiological variables along with temperature and AP values. More research is clearly needed to elucidate the ways in which weather parameters affect stroke incidence, in the hopes that some predictive value, especially for persons already at risk, can be used to decrease stroke events.

## Figures and Tables

**Table 1:** Patient variables.

Demographic	
Age	
Sex	
Ethnicity	
Date of stroke event	
Date/time of emergency department arrival	
Time of event	
Location of event	
Medical/physiological	
Height	
Weight	
Calculated body mass index	
Blood pressure at time of emergency department arrival	
Cholesterol	
Stroke self-history	
Stroke family history	
Diabetes mellitus	
Hypertension	
Other reported conditions in history	
Current medications	
Smoking history	
Alcohol use	
All other drug use and history	
Additional	
NIH Stroke Scale score	
Activity at time of stroke event	
Length of hospital stay	
Disposition at discharge	
Stroke diagnosis	
International Classification of Diseases (9th rev.) code	

**Table 2:** Meteorological variables.

Minimum temperature	
Maximum temperature	
Average temperature	
Average atmospheric pressure	
Hours of sunlight	
Percent average relative humidity	
24-hour precipitation	

**Table 3:** Stroke patient demographics (*N* = 134).

	Subjects, *n* (%)	Age range, y	Average/median age, y
Sex			
Male	58 (43)	35–92	64.10/63.50
Female	76 (57)	20–94	68.40/69.50
Ethnicity			
African American	88 (66)		
White	38 (28)		
Asian	3 (2)		
Native American/Pacific Islander	1 (<1)		
Hispanic	2 (<2)		

**Table 4:** Associations between weather factors and patient variables.

		*P* value
Higher atmospheric pressure		
Stroke history	Y versus N: 1017.28 versus 1015.05 hPA	0.04

Above median atmosphere pressure		
Stroke history	Y versus N: *n* = 34 versus *n* = 14	0.0001
Hypertension	Y versus N: *n* = 61 versus *n* = 41	0.003
Normal BMI	Y versus N: *n* = 18 versus *n* = 8	0.003
Male versus female	*n* = 38 versus *n* = 20	0.04

Above median average temperature		
African American	Y versus N: *n* = 51 versus *n* = 36	0.02
Normal BMI	Y versus N: *n* = 18 versus *n* = 8	0.003
Female versus male	*n* = 44 versus *n* = 31	0.03

BMI: body mass index.
